# Heart Rate Responses during Small Sided Games and Official Match-Play in Soccer

**DOI:** 10.3390/sports4020031

**Published:** 2016-05-30

**Authors:** Alper Aşçı

**Affiliations:** Haliç University School of Physical Education and Sport, İstanbul 34330, Turkey; alperascii@gmail.com; Tel.: +90-533-9290795; Fax: +90-212-2880784

**Keywords:** small sided games, soccer, heart rate, time spent

## Abstract

Small sided games (SSGs) are a match specific type of training. In addition, there is an insufficient number of studies that compare heart rate (HR) responses of SSGs and official match-play (OM). The purpose of this study was to investigate the heart rate responses during SSGs and OM in young soccer players. Twenty-two male soccer players (mean ± SD; age 17.4 ± 0.9 years, height 174.9 ± 6.6 cm, body weight 67.7 ± 8.1 kg) volunteered to participate in this study. The first session included anthropometric measurements and a maximum running test (RT). Following the RT session, all players participated in five different randomly ordered SSG sessions (3-, 4-, 5-, 7- and 9-a-side with goalkeepers). OMs were also monitored in the fourth week of the study. A one-way multivariate repeated-measures analysis of variance (MANOVA) was then conducted to evaluate the differences between the SSGs and OM. The results showed that 3-a-side elicited significantly higher HR and %HR_max_ than other SSGs and OM, whereas 9-a-side resulted in significantly lower HR and %HR_max_ compared to other SSG formats and OM (*p* < 0.05). In conclusion, 3-a-side, 4-a-side and 5-a-side SSG formats provide players with the opportunity to spend sufficient proportion of time spent in high intensity zones that are specific to match demands.

## 1. Introduction

Soccer specific small sided games (SSGs) are often used in soccer training in order to reproduce the physical, technical and tactical requirements of real match-play [1]. SSGs contain many of the elements of soccer match play, such as passing, dribbling skills, and scoring, but typically involve reduced player numbers and/or modified rules [2]. On the technical side, game-specific skill involvements are different during SSGs. Kelly and Drust [3] compared SSG formats according to game-specific skill involvements and found that the number of tackles and shots were higher in SSGs played on smaller pitches than on larger ones. Jones and Drust [4] compared the total number of ball contacts during 4- and 8-a-side games, finding that decreasing player numbers significantly increased the number of individual ball contacts from 13 per game in 8-a-side to 36 in 4-a-side games. Furthermore, game-specific skill involvements are different in SSGs compared to official match play [5]. Capranica *et al.* [5] compared the technical activities of young soccer players in 7-a-side games on a smaller pitch (60 m × 40 m) and in 11-a-side official games on a regular-sized pitch (100 m × 65 m). They found that there were significantly more passes (156 *vs.* 107) in 7-a-side games than in 11-a-side games.

Physiological response during SSGs and match-play has been traditionally determined with the use of heart rate monitors [6,7] because heart rate (HR) measured during soccer exercises effectively reflects the metabolic demands of these intermittent type activities [8]. HR during 5-, 6-, 7- and 8-a-side games has been found to average 169 to 175 bpm [9] corresponding to 85%–90% of maximum heart rate (HR_max_) [9,10,11]. For 3-a-side and 4-a-side games, HR averages 173 to 184 bpm, which corresponds to 90%–95% of HR_max_ [4,9,11,12,13,14]. While HR responses are between 152 to 184 bpm in SSGs (from 1-a-side to 8-a-side) [15,16,17], HR during soccer match play averages 150 to 180 bpm [5,18,19], corresponding to 85% of maximum heart rate (HR_max_) [20].

However, only a few studies have examined the relationship between performance in SSGs and that in competitive matches. Gabbet *et al.* [21] investigated movement patterns in small sided training games (3-a-side and 5-a-side) and compared these movement patterns with those seen in domestic, national, and international standard competitive matches between elite women soccer players. The results of their study suggest that small sided training games simulate the overall movement patterns seen in competitive matches. Dellal *et al.* [22] compared the effects of certain rule changes on the technical and physical demands on elite soccer players in five playing positions in 4-a-side games compared to 11-a-side matches. They found that 4-a-side games played with a rule limiting players to one or two ball touches increased the amount of high-intensity running and the difficulty of performing technical actions, being more specific to match demands. Furthermore, there is no comparative data on physiological responses and proportion of time spent (PTS) in the different heart rate zones to both SSGs and official match-play in male young soccer players. In addition, no study compared physiological responses of 9-a-side and OM. Therefore, this study aims to fill this gap by examining and comparing the heart rate responses and PTS in different heart rate zones during 3-a-side, 4-a-side, 5-a-side, 7-a-side and 9-a-side with similar workout periods and official match play.

## 2. Materials and Methods

### 2.1. Subjects

Fifty-one soccer players in the under 18 (U18) and under 16 (U16) soccer teams in Turkey volunteered to participate in this study. However, twenty-two of them who played 90 min in official matches were included in this study (mean ± SD; age 17.4 ± 0.9 years, height 174.9 ± 6.6 cm, body weight 67.7 ± 8.1 kg, sum of 7-side skinfold thickness 63.1 ± 19.0 mm, maximum heart rate 196.8 ± 7.0 bpm and maximum blood lactate 10.0 ± 2.2 mmol·L^−1^). All players train for one-and-a-half to two hours a day, five times a week, as well as playing official matches on Saturdays or Sundays. All players and parents were notified of the research procedures, requirements, benefits, and risks before giving informed consent. The study was approved by the Hacettepe University Ethics Committee and was conducted in a manner consistent with the institutional ethical requirements for human experimentation in accordance with the Declaration of Helsinki.

### 2.2. Procedures

First of all, the four-week training period served as a familiarization period for the subjects to the SSG formats. After the familiarization period, seven sessions were monitored during a six-week in-season period. Small sided games were performed on Tuesdays. In addition, OM played at the end of the fourth week as well as in the SSGs. Anthropometric measurements and a maximum running test (RT) were assessed in the first session. During the RT, each subject’s heart rate was continuously recorded using heart rate monitors. Following the RT session, all players participated in five different randomly ordered small sided game formats (3-a-side, 4-a-side, 5-a-side, 7-a-side and 9-a-side games, all including the goalkeeper) over the five weeks including one OM per week. No specific rules were introduced to influence the intensity of the SSGs. Average HR, percent of HR_max_ and time spent in heart rate zones (HRZ) corresponding to ≤69%, 70%–84%, 85%–89%, 90%–94% and 95%–100% of maximum heart rate were calculated for all bouts and OM for each subject [23]. The time spent in the respective HRZs were reported as a percentage of the total exercise duration of each SSG and OM.

### 2.3. Field Testing

Each player’s individual HR_max_ and maximal blood lactate (La_max_) was determined using a speed incremental protocol during RT that was performed on an artificial grass soccer pitch. The test included running bouts lasting three minutes and one minute passive rest between bouts. The RT began at a running speed of 8 km·h^−1^, after which the speed was increased to 10 km·h^−1^ and then incremental speed increases of 1 km·h^−1^ were made for each successive running bout following the rest periods until the subjects were exhausted [24]. The subjects adjusted their running speed to auditory signals at 20 m intervals delineated by visual marks along a 100 m track. Capillary blood samples were drawn from the subjects’ earlobes during the one-minute rest periods. Blood lactate was determined using a YSI 1500 Sport Lactate Analyser (Yellow Spring Instruments, Yellow Springs, OH, USA). Subjects’ heart rates (recorded at 5 s intervals) were continuously recorded using heart rate monitors (Polar S-610; Polar Electro OY, Kempele, Finland) during the RT. HR data was stored in the heart rate monitors and downloaded to a computer using Polar Interface Plus with the Polar precision performance software (Version 4.01.029, Polar Electro Oy, Kempele, Finland) at the end of each session. The highest value of recorded heart rates in either of the last two bouts was considered the maximum heart rate (HR_max_).

### 2.4. Small Sided Games and Official Matches

All subjects played games in the five different formats (3-a-side, 4-a-side, 5-a-side, 7-a-side and 9-a-side games), all of which involved the goalkeeper, official-sized goals and an unlimited number of touches on an artificial grass field. The offside rule was not applied during the SSGs [25]. The SSG_S_ were played at the beginning of each training session, following a standardized 20-min warm-up period. Verbal support was given to all subjects during the SSGs to encourage the maintenance of a high work-rate. Players were allowed to consume available drinking water during all recovery periods of the SSGs. Pitch dimensions used (length-width in meters), pitch size (m^2^), pitch size per player (m^2^), the number of bouts, bout duration (min), and work time without rest periods (min) are shown in Table 1. In order to record the HR of each subject in one OM, three official matches in total were monitored at the end of the fourth week of this study as the subjects played on different teams. One OM was played between the first and fourth place of U18 league at 13:30 pm on the Saturday while the other OM was between the second and fifth place of the same league at 13:30 pm on the Sunday. The third OM, a U16 league match, was played between the U16 teams of two first division soccer clubs at 11:00 am on the Sunday. All of these OMs were played on artificial grass pitches. In addition, there was no red card and no injury in the OMs.

Heart rates were continuously recorded at 5 s intervals using short-range radio telemetry (Polar Team Sport System, Polar Electro OY, Kempele, Finland) during the SSGs and OM. Recorded HRs were then downloaded on to a computer using the Team system interface unit following each SGG and OM. Average HR and time spent in zones corresponding to ≤69%, 70%–84%, 85%–89%, 90%–94% and 95%–100% of HR_max_ were calculated for all bouts and for each subject. Proportion of time spent (PTS) in the respective HRZs was calculated by dividing the time spent corresponding to HRZ to the total exercise duration for each SGG and OM. Rest periods between exercise bouts were excluded from the analysis.

### 2.5. Statistical Analysis

Data are expressed as means (M) ± standard deviations (SD). The normality distribution of the data was checked using the Kolmogorov–Smirnov test. A one-way multivariate repeated-measure analysis of variance (MANOVA) was then conducted to evaluate the differences between the SSGs and OM in terms of PTS in each HRZ. HR and %HR_max_ values were analyzed using a one-way repeated-measures analysis of variance. The Bonferroni procedure was applied to make pairwise comparisons among the different-sided games. The level of statistical significance was set at *p* ≤ 0.05. Effect sizes (η^2^) were also calculated and values of 0.01, 0.05 and above 0.15 were considered small, medium and large, respectively [26]. All statistical analysis was carried out using SPSS statistical software (SPSS Version 15.0 for Windows, Chicago, IL, USA).

## 3. Results

**Figure 1:**
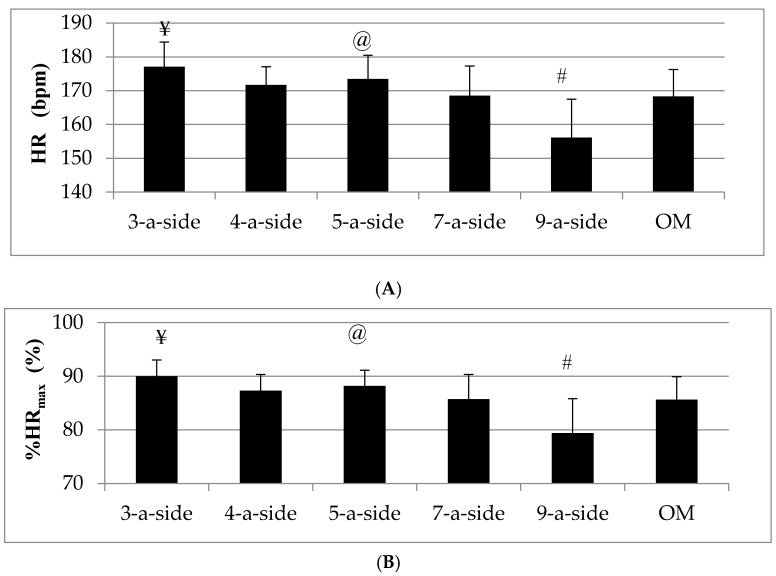
Mean HR (**A**) and %HR_max_ (**B**) during small sided games (SSGs) and official match (OM). HR: Heart rate; ¥ significantly different from other SSGs formats and OM, *p* < 0.05; @ significantly different from 7-a-side game, *p* < 0.05; # significantly different from all other SSGs formats and OM, *p* < 0.05.

Figure 1 shows the mean ± standard deviation frequency of HR and %HR_max_ values during SSGs and OM. The lowest HR and %HR_max_ responses (156.1 ± 11.4 bpm, 79.4% ± 6.4%, respectively) were found in 9-a-side game, while the 3-a-side game resulted in the highest HR and %HR_max_ responses (177.1 ± 7.3 bpm, 90.0% ± 3.0%, respectively). One-way repeated ANOVA showed statistical significant differences among SSGs and OM in terms of HR (F = 25.057; *p* = 0.001; η^2^ = 0.544; large effect) and %HR_max_ (F = 25.594; *p* = 0.001; η^2^ = 0.549; large effect). *Post hoc* pairwise comparisons revealed that 3-a-side elicits significantly higher HR and %HR_max_ responses than other SSGs and OM, whereas 9-a-side elicits significantly lower HR and %HR_max_ responses compared to other SSGs and OM.

Table 2 shows the mean PTS (± SD) in different HRZs for each SSG format as well as for the OM. One-way repeated ANOVA showed statistical significant differences among SSGs and OM in terms of ≤69% of HRZ (F = 5.242; *p* = 0.001; η^2^ = 0.200; large effect), 70%–84% of HRZ (F = 40.913; *p* = 0.001; η^2^ = 0.661; large effect), 85%–89% (F = 2.644; *p* = 0.027; η^2^ = 0.112; medium effect), 90%–94% (F = 23.805; *p* = 0.001; η^2^ = 0.531; large effect) and 95%–100% of HRZ (F = 6.190; *p* = 0.001; η^2^ = 0.228; large effect). *Post hoc* pairwise comparisons revealed that 3-a-side had a significantly lower PTS in the under ≤69% of HRZ than other SSG formats. In addition, a 9-a-side game and OM had significantly higher PTS in the 70%–84% of HRZs than all other SSGs. Moreover, 7-a-side had significantly higher PTS in the 85%–89% of HRZ compared to OM. Furthermore, 9-a-side games had significantly lower PTS in the 90%–94% of HRZ than all other SSGs and OM. In addition, OM had significantly lower PTS in the 90%–94% of HRZ than in 3-a-side. Lastly, 9-a-side had significantly lower PTS in the 95%–100% of HRZ than OM and all other SSG formats with the exception of 7-a-side.

## 4. Discussion

The purpose of this study was to investigate the heart rate responses during SSGs and OM in young soccer players. In this study, different formats of SSGs were used when compared the SSGs with a different number of players and OM. The main findings show that 3-a-side, 4-a-side and 5-a-side SSG formats provide players with the opportunity to spend sufficient PTS in high intensity zones, while 7-a-side and 9-a-side SSG formats provide players with the opportunity to spend sufficient PTS in low intensity zones, which are specific to match demands.

Hoff *et al.* [7] found HR and %HR_max_ to be a reliable way of establishing the exercise intensity carried out in small sided games. Therefore, in the present study, exercise intensity is determined by heart rate and percent of maximum heart rate during 3-a-side, 4-a-side, 5-a-side, 7-a-side, 9-a-side and OM. Mean %HR_max_ value of SSGs ranged between 79%–90% in the current study. These values are similar to previous studies [9,12,27,28]. In addition, the results of this study show that 3-a-side elicits significantly higher HR and % HR_max_ responses than other SSG formats and OM, whereas 9-a-side elicits significantly lower HR and percent of HR_max_ responses compared to other SSG formats and OM. The present study also shows that 5-a-side elicits significantly higher HR and %HR_max_ responses than 7-a-side. Little and Williams [9] found that, as the pitch size and the number of players increase in SSGs, HR and %HR_max_ responses of English professional soccer players decrease. Parallel to findings of Little and Williams [9], the current study shows that when the pitch size per player is increased, the intensity and the involvement into the SSGs might be decreased with the exception of 9-a-side *versus* OM. The reason may be due to different total work duration of these games. OM was played 90 min while total duration of 9-a-side game was played 16 min. Therefore, the increase of game duration may have caused to an increase in heart rate responses in OM compared to 9-a-side game.

This study shows that 9-a-side resulted in significantly lower PTS than 3-a-side, 4-a-side, 5-a-side and 7-a-side games and OM in the high intensity zone, which is 90%–94% of HRZ. In addition, PTS in a very high intensity zone, which is 95%–100% of HRZ, was similar in OM and the SSGs with the exception of 7-a-side and 9-a-side games. Thus, the present study shows that 3-a-side, 4-a-side, 5-a-side games allow match specific high intensity loads. When the pitch size per player is increased, the intensity and the involvement into the SSGs might be decreased. In addition, reductions in the number of players have also been found to increase the number of technical actions such as ball contacts [4,29]. In addition, Jones and Drust [4] reported that backwards and sideways movements increase when the number of the players is decreased. Therefore, backwards and sideways movements may be a possible reason of spending more time in higher HRZ in SSGs when the number of players is decreased. Parallel to the present study results, Owen *et al.* [29] found that 3-a-side games elicited significantly higher exercise intensity in terms of a greater amount of time spent in intense HRZ compared to 9-a-side games. Hill-Haas *et al.* [30] found that the 2-a-side format elicited a greater amount of time spent above 90% HR_max_ than the 4-a-side and 6-a-side formats.

There are some limitations of the present study. One of these limitations, unfortunately, involved not being able to measure the distance covered at various running speeds and technical actions, and, therefore, future studies that investigate time-motion analysis and technical actions during the SSGs and OM are needed. In addition, another limitation of this study was not equal for some variables such as pitch size, pitch size per players, number of bouts, bouts duration and total time in SSGs. For example, players performed 16 min for 9-a-side game, while players performed 12 min for all the other SSGs.

## 5. Conclusions

Little [2] stated that SSGs that involve more players, such as 5-a-side, 6-a-side, 7-a-side, and 8-a-side games, can be used to develop the anaerobic threshold (85%–90% of HR_max_), whereas 3-a-side and 4-a-side games might be used with the aim of enhancing maximum oxygen consumption. The results of this study suggest that 3-a-side, 4-a-side and 5-a-side games could be used for high intensity soccer specific aerobic endurance training. However, if coaches want higher %HR_max_ responses and PTS in high intensity zones, they should be organize 3-a-side games. In addition, 7-a-side and 9-a-side games have lower HR responses compared to OM. Therefore, 7-a-side and 9-a-side games seem to not be suitable for high intensity training principles. Finally, 7-a-side and 9-a-side games may be used for low intensity aerobic training by coaches.

## Figures and Tables

**Table 1:** Characteristics of the small sided games including goalkeepers and official match (OM).

Games	Pitch Dimension (m)	Pitch Size (m^2^)	Pitch Size per Player (m^2^/player)	Number of Bouts	Bout Duration (min)	Total Time Without Rest Periods (min)
3-a-side	25 × 20	500	83	6	2	12
4-a-side	35 × 30	1050	131	4	3	12
5-a-side	45 × 30	1350	135	3	4	12
7-a-side	55 × 40	2200	157	2	6	12
9-a-side	70 × 40	2800	155	2	8	16
OM	105 × 65	6825	341	1	90	90

**Table 2:** Mean proportion of time spent (%) in respective heart rate zones during small sided games and official match (± SD).

Zones	Games					
	3-a-Side	4-a-Side	5-a-Side	7-a-Side	9-a-Side	OM
**95%–100%**	14.2 ± 18.2 ^§^	13.6 ± 16.1 ^§^	16.0 ± 17.9 ^§^	4.9 ± 10.6	1.2 ± 3.0	12.9 ± 11.5 ^§^
**90%–94%**	48.1 ± 20.8 ^§^	37.3 ± 17.9 ^§^	35.9 ± 17.7 ^§^	35.3 ± 17.3 ^§^	7.9 ± 10.0	24.9 ± 10.0 *^,§^
**85%–89%**	21.3 ± 14.7	22.8 ± 14.2	25.2 ± 15.3	32.9 ± 15.3 ^¥^	20.8 ± 15.7	22.1 ± 5.7
**70%–84%**	14.6 ± 7.8 ^§,¥^	18.7 ± 11.7 ^§,¥^	17.8 ± 10.5 ^§,¥^	17.8 ± 16.2 ^§,¥^	54.5 ± 18.3 ^¥^	31.9 ± 12.2 ^§^
**≤69%**	1.7 ± 2.5	7.6 ± 6.1 *	5.0 ± 2.9 *	7.3 ± 6.8 *	15.0 ± 17.1 *	8.2 ± 12.2

OM: Official match; * significantly different from 3-a-side game, *p* < 0.05; ^§^ significantly different from 9-a-side game, *p* < 0.05; ^¥^ significantly different from OM, *p* < 0.05.
